# Commentary: Global prevalence and antibiotic resistance in clinical isolates of *Stenotrophomonas maltophilia*: a systematic review and meta-analysis

**DOI:** 10.3389/fmed.2024.1340358

**Published:** 2024-01-31

**Authors:** Emad Heydarnia, Nahal Majidzadeh, Behnaz Seyedin, Vafa Saber

**Affiliations:** ^1^Department of Medical Nanotechnology, Faculty of Advanced Technologies in Medicine, Iran University of Medical Science, Tehran, Iran; ^2^Faculty of Dentistry, Medical University of Pécs, Pécs, Hungary; ^3^Department of Microbiology, Azad University, Shahr-e Qods, Tehran, Iran; ^4^Biochemistry and Biophysics Research Center, North Tehran Azad University, Tehran, Iran

**Keywords:** *Stenotrophomonas maltophilia*, methodology, antibiotic resistance, meta-analysis, prevalence

## Introduction

We have thoroughly reviewed the article titled “Global prevalence and antibiotic resistance in clinical isolates of *Stenotrophomonas maltophilia*: a systematic review and meta-analysis,” investigating the global prevalence and antibiotic resistance of *S. maltophilia* (1). The study conducted searches in MEDLINE (via PubMed), Embase, Web of Science, and Scopus, utilizing diverse keywords until October 20, 2019, with a focus on clinical isolates and applying rigorous exclusion criteria for relevance.

However, our examination has revealed critical methodological concerns that impact the precision of the global epidemiological insights into *S. maltophilia* and its antibiotic resistance patterns. In the subsequent sections, we outline these concerns and suggest recommendations for refinement.

The primary objective of the study was to evaluate the global prevalence of *S. maltophilia* and its resistance to commonly used antibiotics. The methodology involved searches across specified databases using a variety of keywords, targeting clinical isolates and applying exclusion criteria to eliminate environmental isolates. Articles were included based on their reporting of *S. maltophilia* prevalence among diverse patients, either in conjunction with antibiotic resistance rates or reporting resistance rates alone. Articles focusing solely on resistant isolates or reporting only prevalence were excluded.

Several issues potentially introduce bias into the study results. In systematic reviews and meta-analyses, comprehensive searches are imperative to collect the most pertinent data. Regrettably, the search syntax used in this study does not adequately align with the primary objective of investigating the prevalence of *S. maltophilia* isolates among diverse patients. Furthermore, the exclusion of articles reporting only on the prevalence of *S. maltophilia* conflicts with the study's central outcome.

Concerning the secondary objective of assessing antibiotic resistance prevalence, the inclusion of keywords such as “multilocus sequence typing” and “antimicrobial resistance gene” is incongruent with the study's objectives and poses limitations on the search. Additionally, the inclusion criteria defining the population as diverse patients are suitable for antimicrobial resistance but may introduce selection bias for *S. maltophilia* prevalence. Some studies report a 100% prevalence for *S. maltophilia*, and the lack of a clear population definition contributes to this selection bias, potentially involving pre-diagnosed patients.

The study lacks a precise definition of antibiotic resistance, leading to potential bias in the absence of resistance isolate definitions. Recommendations for improvement encompass refining the search strategy, modifying inclusion and exclusion criteria, providing clear meta-analysis definitions, and conducting separate analyses based on antibiotic breakpoints or established guidelines.

## Conclusion

In conclusion, this commentary underscores critical methodological concerns within the published study and emphasizes the necessity for refining the methodology to ensure a more accurate depiction of global *S. maltophilia* epidemiology and antibiotic resistance patterns. We recommend reconsidering the focus on antibiotic resistance rates or, if retaining prevalence, including all relevant studies through a revision of the search syntax and criteria. Clear definitions for the study population and resistance, along with separate analyses based on different antibiotic breakpoints or guidelines, are essential for methodological robustness.

## Author contributions

EH: Investigation, Supervision, Writing—original draft. NM: Supervision, Writing—original draft. BS: Supervision, Writing—original draft. VS: Conceptualization, Investigation, Project administration, Resources, Supervision, Validation, Writing—original draft.

## References

[1] Banar M Sattari-Maraji A Bayatinejad G Ebrahimi E Jabalameli L Beigverdi R. Global prevalence and antibiotic resistance in clinical isolates of *Stenotrophomonas maltophilia*: a systematic review and meta-analysis. Front Med. (2023) 10:1163439. 10.3389/fmed.2023.1163439 37215718 PMC10196134

